# Adaptation of a Personalized Electronic Care Planning Tool for Cancer Follow-up Care: Formative Study

**DOI:** 10.2196/41354

**Published:** 2023-01-10

**Authors:** Stephanie J Sohl, Pamela W Duncan, Elyse Thakur, Nicole Puccinelli-Ortega, John M Salsman, Greg Russell, Boris C Pasche, Stacy Wentworth, David P Miller Jr, Lynne I Wagner, Umit Topaloglu

**Affiliations:** 1 Wake Forest University School of Medicine Winston-Salem, NC United States; 2 Atrium Health Wake Forest Baptist Comprehensive Cancer Center Winston-Salem, NC United States; 3 Atrium Health Charlotte, NC United States

**Keywords:** cancer survivorship, cancer survivor, colorectal cancer, patient engagement, shared decision-making, follow-up care, patient care planning, electronic tool, patient-reported outcomes, behavior change

## Abstract

**Background:**

Most patients diagnosed with colorectal cancer will survive for at least 5 years; thus, engaging patients to optimize their health will likely improve outcomes. Clinical guidelines recommend patients receive a comprehensive care plan (CP) when transitioning from active treatment to survivorship, which includes support for ongoing symptoms and recommended healthy behaviors. Yet, cancer care providers find this guideline difficult to implement. Future directions for survivorship care planning include enhancing information technology support for developing personalized CPs, using CPs to facilitate self-management, and assessing CPs in clinical settings.

**Objective:**

We aimed to develop an electronic tool for colorectal cancer follow-up care (CFC) planning.

**Methods:**

Incorporating inputs from health care professionals and patient stakeholders is fundamental to the successful integration of any tool into the clinical workflow. Thus, we followed the Integrate, Design, Assess, and Share (IDEAS) framework to adapt an existing application for stroke care planning (COMPASS-CP) to meet the needs of colorectal cancer survivors (COMPASS-CP CFC). Constructs from the Consolidated Framework for Implementation Research (CFIR) guided our approach. We completed this work in 3 phases: (1) gathering qualitative feedback from stakeholders about the follow-up CP generation design and workflow; (2) adapting algorithms and resource data sources needed to generate a follow-up CP; and (3) optimizing the usability of the adapted prototype of COMPASS-CP CFC. We also quantitatively measured usability (target average score ≥70; range 0-100), acceptability, appropriateness, and feasibility.

**Results:**

In the first phase, health care professionals (n=7), and patients and caregivers (n=7) provided qualitative feedback on COMPASS-CP CFC that informed design elements such as selection, interpretation, and clinical usefulness of patient-reported measures. In phase 2, we built a minimal viable product of COMPASS-CP CFC. This tool generated CPs based on the needs identified by patient-completed measures (including validated patient-reported outcomes) and electronic health record data, which were then matched with resources by zip code and preference to support patients’ self-management. Elements of the CFIR assessed revealed that most health care professionals believed the tool would serve patients’ needs and had advantages. In phase 3, the average System Usability Scale score was above our target score for health care professionals (n=5; mean 71.0, SD 15.2) and patients (n=5; mean 95.5, SD 2.1). Participants also reported high levels of acceptability, appropriateness, and feasibility. Additional CFIR-informed feedback, such as desired format for training, will inform future studies.

**Conclusions:**

The data collected in this study support the initial usability of COMPASS-CP CFC and will inform the next steps for implementation in clinical care. COMPASS-CP CFC has the potential to streamline the implementation of personalized CFC planning to enable systematic access to resources that will support self-management. Future research is needed to test the impact of COMPASS-CP CFC on patient health outcomes.

## Introduction

Colorectal cancers are among the most prevalent cancers, with over 1.4 million men and women living with a prior diagnosis of colorectal cancer [1]. Approximately 66% of colorectal cancer patients will survive for at least 5 years after the diagnosis [2]; however, many cancer patients get lost in the transition from active cancer treatment to survivorship and do not receive the guidance they need to manage lasting effects (eg, fatigue, cognitive difficulties, and pain) and increased health risks (eg, cardiac problems and secondary malignancies [3,4]). Thus, there is an opportunity to improve cancer follow-up care (CFC), including providing support for how survivors can take a more active role to optimize their own health outcomes (ie, self-management) [3].

One way in which CFC can be optimized is through comprehensive care plans (CPs), which are recommended in clinical guidelines [3,5]. These plans outline follow-up care needs, including support for lasting effects (eg, symptom control) and recommendations for lifestyle changes or health behaviors to reduce health risks [3,5,6]. Historically, such plans have focused on information delivery, which had minimal benefit [5,7]. Other barriers to effective care planning included time and resources to develop CPs, lack of routine assessment of patient-reported outcome (PRO) measures to guide symptom management, and complexity of shifting the paradigm of care from diagnosis and treatment to minimizing long-term risks [4,7]. To improve clinical outcomes, recommendations include using technology-based solutions to support follow-up care planning as a personalized and ongoing process [4], in addition to providing resources to promote self-management. To empirically assess these benefits, it was recommended that care planning interventions be evaluated with an implementation science approach in real-world clinical settings [4,5,7-9].

To address this gap in the literature, we customized an electronic care planning tool we previously implemented for a large pragmatic trial of an intervention (COMPASS-CP) designed to support stroke care in the real-world setting [10-12]. COMPASS-CP captures and integrates patient-completed data (including validated PROs, social determinants of health, and assessments of health behaviors) with clinical data via a standard-based integration of electronic health record (EHR) data to enable provider-facilitated decision-making to immediately generate personalized CPs [10]. Its questionnaires are designed to yield a comprehensive overview of factors that can impair a patient’s ability to manage his or her health and recovery. COMPASS-CP also houses a directory of medical, rehabilitation, and community resources to support identified concerns, which can be used to address identified needs, thus making CPs actionable to improve the patient’s function and quality of life. Moreover, integration with the EHR (Epic) in an interoperable manner using the Substitutable Medical Applications and Reusable Technologies (SMART) on Fast Health Interoperability Resources (FHIR) standard promotes scalability. This unique combination of features is not currently present in most other electronic survivorship care planning tools or the EHR itself [13-15]. For example, a similar tool links patient data to create a CP with guideline-concordant guidance and not community resources [16]. Participants who successfully received an intervention that included this electronic care planning tool (COMPASS-CP) had better functional status and satisfaction with care than those who did not [17]. Stroke health care providers (n=44) indicated high user satisfaction (eg, 74% agreed that the electronic tool identified important factors impacting the patient’s recovery and ability to self-manage) [10].

The success of the COMPASS-CP tool prompted us to consider other populations that may benefit from a similar resource, such as those diagnosed with cancer. This tool has the potential to compile the core components of CFC planning. Facilitating personalized links to community resources as a component of survivorship care planning is aligned with the need for community support during this process [18]. The identification of such community resources is especially important for rural populations as they may not have access to resources available in larger metropolitan areas. With limited access to resources, patients in such settings have higher rates of health behavior–related risk factors (ie, age-adjusted smoking and obesity), cancer incidence, and mortality, as compared with the rest of the population [19-22]. These patients may have decreased compliance with recommended follow-up. Thus, it is important to consider how to best develop comprehensive and personalized CPs that consider pertinent risk factors for this patient population.

This study adapted the COMPASS-CP tool to colorectal CFC planning (COMPASS-CP CFC) with a focus on generating the follow-up CP component (ie, integrating and not changing the treatment summary already used in clinical care). We adopted a user-centered design process to adapt COMPASS-CP and evaluated elements in the Consolidated Framework for Implementation Research (CFIR) [23] to plan for successful implementation in clinical care. In order to prepare for implementing this tool into a clinical workflow to facilitate comprehensive care, we first gathered input from key stakeholders. Patient input on survivorship care needs was also gathered through our previous work [24]. We then adapted assessments, algorithms, and resource data sources needed to generate a cancer follow-up CP. Lastly, we tested the usability of the adapted prototype version of COMPASS-CP CFC. 

## Methods

### Study Design

To adapt COMPASS-CP to colorectal cancer survivors who completed treatment, we adopted the Integrate, Design, Assess, and Share (IDEAS) framework, which consists of the following 10 stages: (1) empathize with target users; (2) specify the target behavior; (3) ground in behavioral theory (self-determination theory to inform future work [25]); (4) ideate implementation strategies; (5) prototype potential products; (6) gather user feedback; (7) build a minimum viable product, defined as a fully functional initial intervention with only the most essential features; (8) pilot test to assess potential efficacy and usability (current and future studies); (9) evaluate efficacy (future studies); and (10) share the intervention and findings (future studies) [26]. Stages 1-3 are part of the Integrate aspect, stages 4-7 are part of the Design aspect, stages 8 and 9 are part of the Assess aspect, and stage 10 is part of the Share aspect of the framework. This framework is implemented iteratively until a refined viable product is developed [27,28]. We also used a mixed methods approach to assess implementation outcomes first in focus groups and then after building a minimum viable product as part of usability testing.

### Ethical Considerations

Study procedures involving the participation of human subjects received institutional review board (IRB) approval (phase 1: IRB00059620; phase 3: IRB00064181). Participants were provided an information sheet that described study involvement. This sheet stated, “By continuing, I agree to take part in this study,” with a waiver for signed consent. Study data were deidentified. Patients and caregivers received US $50 for participation in phase 1. 

### Description of the COMPASS-CP CFC Process

**Figure 1:**
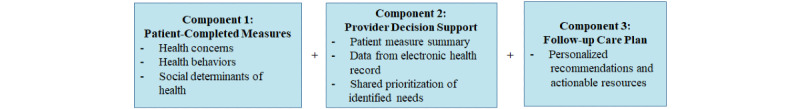
Summary of COMPASS-CP CFC application components. CFC: cancer follow-up care; CP: care plan.

Patients who completed treatment of colorectal cancer and health care professionals involved with colorectal cancer care were targeted for this application development. The study workflow is shown in Figure 1. The first component involved patients filling out measures before a clinic visit to inform provider sessions. Completion of the provider decision support tool (component 2) then facilitated shared decision-making and resulted in real-time creation of a personalized follow-up CP (component 3).

### Phase 1: Learning From Target Users, Ideating Implementation Strategies, and Gathering Feedback on Prototype Potential Products

#### Focus Groups

We elicited feedback to iteratively refine the cancer follow-up CP generation design and workflow. First, we conducted two 60-minute focus groups (one with patients and caregivers, and one with health professionals) for feedback on content displayed on paper prototypes (enlarged screenshots of application content) with a divergent brainstorming focus (IDEAS stages 1, 4, 5, and 6). 

The first patient and caregiver focus group reviewed application components 1 and 3. REDCap (Vanderbilt University, TN), an established web-based application, was adopted as the patient-facing interface for completing surveys. Questionnaire data stored in REDCap were retrieved by COMPASS-CP electronically via application programming interfaces (APIs). REDCap surveys can be sent to participants through a variety of methods, including a unique link (ie, token), which will take them directly to the survey without the need to log in to complete the survey. Thus, feedback was mostly requested on how to make the resulting CP actionable. Patient eligibility criteria included the following: age ≥18 years; diagnosis of stage I-III colorectal cancer; cognitive ability to complete interviews; and ability to understand, read, and write English. Caregiver eligibility criteria included the following: age ≥18 years and provision of some capacity of unpaid care for a patient meeting the patient eligibility criteria.

The second health care professional focus group (providers, administrators, and informatics professionals) reviewed application components 2 and 3 within the context of an example patient case. Health care professionals included key representatives from our study team.

#### Semistructured Interviews

We then conducted semistructured interviews for another round of feedback with the health care professional participants (some were follow-up interviews with stakeholders who participated in the focus groups and some were initial interviews with additional stakeholders). These interviews had a convergent focus and identified anything that was missing or needed to be refined to ensure integration of COMPASS-CP CFC into standard care. Both rounds of feedback were audio recorded, and the follow-up interviews were transcribed verbatim by an external service.

The follow-up interviews included questions guided by the CFIR [23] to facilitate assessment of potential barriers and facilitators at multiple levels and help us prepare for future implementation of the COMPASS-CP CFC tool. *Process components* of this framework guided our planning and engagement of opinion leaders (The Wake Forest Baptist Comprehensive Cancer Center leadership, oncologists, and information technology professionals), implementation leaders (advanced practice providers and navigators), champions (study team and others), and possible external change agents (clinical guidelines and requirements) to prepare for future implementation. Engagement was iterative and incorporated into the application development to strengthen stakeholders’ representation. Interview questions relevant to this stage of research asked about the *inner setting* constructs of culture, implementation climate, learning climate, and leadership engagement and the *outer setting* constructs of patient needs and resources, perceived influence of external policy and incentives for implementation of survivorship care planning, and potential peer pressure to innovate regarding how to address these policies [29].

### Phase 2: Clarifying the Target Behavior and Building a Minimum Viable Product

We iteratively compiled feedback to clarify the most important data elements to incorporate in creating follow-up CPs (IDEAS stages 2 and 7). The core set of data elements in relevant clinical guidelines for follow-up care of colorectal cancer included the following [14,30-32]: (1) schedule of clinic visits; (2) cancer surveillance or other recommended tests (eg, surveillance colonoscopy, history, and physical screening for other primary cancers); (3) assessment and management of physical and psychosocial long-term and late effects of colorectal cancer and its treatment (eg, bowel/gastrointestinal issues, numbness/tingling, and ostomy/stoma); (4) other late and long-term effects (depression, anxiety, fatigue, sleep disturbance, sexual function, fertility, financial toxicity, insurance, memory/cognitive function, social functioning, physical functioning, and pain); (5) health behaviors (alcohol use, diet/nutrition, obesity/weight changes, physical activity, smoking, and sunscreen use); and (6) care coordination and practice implications (communication among specialists, management of medications, and management of other illnesses). The development team evaluated the availability of the identified data elements in Epic.

COMPASS-CP currently uses SMART on FHIR, which is a data exchange standard to enhance interoperability with EHRs and other systems. The FHIR Patient Resource pushes the patient medical record number and other demographics into COMPASS-CP, which creates the patient record. It is also possible through additional FHIR resources (eg, Encounter) for COMPASS-CP to be able to communicate back the CPs and provider reports to Epic.

We identified appropriate patient-completed measures for assessing other essential data elements. To streamline the integration of the patient-completed data in this study, we integrated the REDCap electronic data capture system. Therefore, patients were able to complete assessments independently (prior work was conducted using a phone interview). REDCap has a comprehensive and publicly accessible library of PRO measures. COMPASS-CP uses REDCap’s extensive APIs, which enable bidirectional data exchange in a secure and scalable manner, to incorporate PRO data and other patient-completed data for CP generation.

We created algorithms in COMPASS-CP CFC informed by clinical guidelines [32] to integrate patient-completed measures with provider-facilitated decisions and immediately generate personalized CPs. We also updated the existing COMPASS-CP resource database to be more specific to cancer care [32]. The minimal viable product of COMPASS-CP CFC generated CPs based on the needs identified by elevated health concerns raised in the patient-completed measures and EHR data elements, which were then matched with relevant resources that could be selected by zip code and preference to support self-management. Patient zip code from the FHIR drives the filtering and recommendation of proper local community resources that are geographically close to the patient.

### Phase 3: Assessing the Usability of COMPASS-CP CFC

We individually implemented the minimal viable version of COMPASS-CP CFC and elicited qualitative feedback about the potential efficacy and usability (IDEAS stage 8). We conducted 2 rounds of usability tests and expected to reach saturation with 5 participants per round [33,34]. The first round of testing was with health care professionals (informatics professionals and health care providers) using mock patient scenarios. For the second round, a health care professional met with patients who were colorectal cancer survivors. Both rounds of feedback used a concurrent “think aloud” protocol and retrospective probing questions regarding acceptability [35]. We used the System Usability Scale and aimed to achieve an average score ≥70 (considered “good”) [34,36,37]. We further assessed quantitative measures of acceptability, appropriateness, and feasibility (Acceptability of Intervention Measure, Intervention Appropriateness Measure, and Feasibility of Intervention Measure, respectively) [38]. 

We also incorporated qualitative assessments in a brief semistructured interview and questionnaire after the think-aloud testing in both groups [39]. With the relevant components of the CFIR Qualitative Interview guide, we systematically considered select* intervention characteristics* (design quality and packaging) and *individual characteristics* (knowledge and beliefs about the intervention) to inform the future content, skills training, and support needed by those who will be using the application. 

### Analyses

Detailed field notes and audio recordings were reviewed to summarize qualitative data regarding participant recommendations that were iteratively incorporated into the application adaptation. For the CFIR analyses to inform future implementation, we reviewed all deidentified transcripts by listening to the audio while reading the transcripts to ensure accuracy. Transcripts were imported into ATLAS.ti (version 7.5; ATLAS.ti Scientific Software Development GmbH) to manage the data. Two research team members independently coded each transcript using the CFIR codebook. After the data were coded, the data within each category were abstracted and synthesized into themes. Themes were determined by their prevalence and salience in the data, according to the principles of thematic analysis [40]. Participant characteristics and usability data were summarized using descriptive statistics.

## Results

### Phases 1 and 2: Gathering and Incorporating Stakeholder Feedback

The characteristics of health care professionals who provided feedback are described in Table 1, and the characteristics of patients and caregivers are described in Table 2. The first focus group included 3 health care professionals, and this was followed up by 7 individual interviews to represent feedback from 2 nurses, 2 nurse practitioners, 1 physician, 1 physician assistant, and 1 allied health professional who was in an administrative role. The second focus group included 5 patients and 2 caregivers.

**Table 1:** Demographic characteristics of health care professionals.

Characteristic			Phase 1 (N=7)	Phase 3 (N=5)	
**Sex, n (%)**					
	Female	7 (100)			2 (40)
	Male	0 (0)			3 (60)
**Ethnicity, n (%)**					
	Not Hispanic or Latino	7 (100)			5 (100)
**Race, n (%)**					
	White	7 (100)			5 (100)
Age (years), mean (SD)			44 (14)	46 (11)	

**Table 2:** Characteristics of colorectal cancer survivors and caregivers.

Characteristic			Phase 1 (N=7)	Phase 3 (N=5)
**Sex, n (%)**				
	Female	4 (57)		3 (60)
	Male	3 (43)		2 (40)
**Ethnicity, n (%)**				
	Not Hispanic or Latino	7 (100)		5 (100)
**Race, n (%)**				
	White/Caucasian	3 (43)		4 (80)
	Black/African American	2 (29)		0 (0)
	Asian	2 (29)		1 (20)
**Marital status, n (%)**				
	Married	5 (71)		3 (60)
	Separated/never married	2 (29)		2 (40)
**Education, n (%)**				
	High school or equivalent	2 (29)		1 (20)
	Some college/technical	4 (57)		0 (0)
	College graduate/postgraduate	1 (14)		4 (80)
**Occupational status, n (%)**				
	Employed	3 (43)		1 (20)
	Self-employed/homemaker	2 (29)		2 (40)
	Retired	0 (0)		2 (40)
	Disabled	2 (29)		0 (0)
**Income (US$), n (%)**				
	0-19,999	3 (43)		0 (0)
	20,000-49,999	2 (29)		2 (40)
	50,000-99,000	1 (14)		1 (20)
	≥100,000	1 (14)		2 (40)
Age (years), mean (SD)		50 (10)		60 (14)

#### Selection of Patient-Completed Measures

We iteratively incorporated stakeholder feedback on the relative importance of recommended data elements and how to best assess them. For example, we asked health care professionals to indicate which patient-completed measures would be most important to evaluate with rigor, briefly, or not at all. Those prioritized for more rigorous patient assessment were assessed with Patient-Reported Outcomes Measurement Information System (PROMIS) measures (ie, anxiety, depression, fatigue, and physical function), as were other constructs that were also captured by the PROMIS-29 profile measure already in use clinically by our local cancer survivorship care providers (ie, social functioning, pain, and sleep disturbance). The PROMIS measures were also used for brief assessments when the clinical cutoffs were clear and the number of items was similar to other assessments (also adopted for assessing cognitive function and sexual function) [41,42]. We adopted PRO-CTCAE (Patient-Reported Outcomes version of the Common Terminology Criteria for Adverse Events; another widely used measurement system specifically for cancer-related symptomatic toxicity) [43] items for other brief assessments of colorectal cancer–specific health concerns (numbness and tingling [1 item for severity and 1 item for interference], and presence of an ostomy appliance). We grouped together these 3 items specific to colorectal cancer and expected all other items assessed to generalize more broadly to CFC. We screened for health behaviors with items from assessments already used clinically for smoking [44] and alcohol use [45], and with other screening instruments for diet [46] and physical activity [47,48].

We also broadened the concept of social functioning to include relevant social determinants of health previously identified in the initial study of COMPASS-CP. This included a measure of health literacy [49], presence of a caregiver, desire for support creating a living will, family and community support [48], and items already included in the EHR record to assess financial, food, and transportation challenges [48]. Furthermore, the original COMPASS-CP for stroke care included an item to briefly assess “What really matters to you and is a reason for you to be optimally healthy?” that we adapted from a values exercise [50]. Examples included in the description of each value option provided were from prior work with cancer survivors [24].

Other variables were extracted directly from the EHR, including date of birth, birth sex (incorporated into logic regarding recommendations for alcohol use), height, weight (used to calculate BMI to inform recommendations regarding weight management), and zip code (to inform resources provided).

#### Additional Stakeholder Feedback

Examples of other feedback that informed adaptations included offering the patient-completed measures in multiple formats (paper and electronic) and allowing patients to choose the most acceptable format. Patients said they would expect a provider to use the information collected from patient-completed measures to identify what is most important to talk about during the clinical encounter. This was a change from the original COMPASS-CP such that we adopted a self-report patient assessment format (rather than a facilitated interview), which particularly impacted our choice of cognitive assessment. A self-reported cognitive assessment was considered more appropriate for cancer survivors than neuropsychological tests administered by the study team because stakeholder feedback indicated that we should include only a brief cognitive assessment, and cognitive difficulties specific to cancer treatment are often too subtle for neuropsychological tests. Other feedback included that provider decision support should clearly indicate thresholds to help the interpretation of results. Multiple other specific modifications were suggested and incorporated.

Positive feedback about the CP from health care professionals included that they liked that it would be personalized and would not contain extraneous information. Furthermore, stakeholders suggested that the resource linking tool should include headings that explain what each resource will address. They also appreciated the feature that allowed searching for resources by zip code, given that help in identifying resources for patients who are not local is an unmet need for survivorship care providers.

#### Summary of CFIR Outer Setting

##### Patient Needs and Resources

Health care professionals were asked to discuss how well they believed the tool would meet the needs of individuals served by the organization. Most said it would serve the needs of patients because it will consolidate survivorship resources for them in the areas where they reside, whereas 1 health care professional thought it was oversimplified (eg, could do more to normalize distress).

##### External Policies and Incentives

Health care providers suggested financial and other incentives that would influence implementation and affect revenue such as increased internal referrals, adding personalized care, seeing more patients who are attracted to the service, conducting joint services (eg, including life coaches or social workers), and saving provider time. One provider was concerned about fulfilling the responsibility to provide adequate and timely resources for a patient who scores high for depression or other relevant issues. One stakeholder commented as follows:

Saving time is really important. I think we can see more patients and we cannot have to follow up a lot after the visit, as much, with our own researching. Trying to find resources for patients and following up with them later. Doing it right there on the spot during their visit would be really helpful and would allow us to see more patients.

Participant ID 003

##### Peer Pressure

Health care professionals were asked how implementing the tool would provide an advantage to the local cancer center compared with other organizations and if there was something specific that would attract more patients. Most said the tool may provide an advantage to the organization by being known for providing wellness resources to assist patients in their own communities.

#### Summary of CFIR Inner Setting

##### Culture

When asked to what extent new ideas are embraced and used to make improvements in the organization, most health care professionals said new ideas are embraced in their immediate area; however, the organization as a whole is slower to embrace new ideas.

##### Implementation Climate: Tension for Change

Health care professionals were asked if they believed there was a strong need for the tool. Most participants said they saw a need for the survivorship CP tool because survivorship is growing, and the tool provides for a “survivorship pathway,” removes burden from providers, and provides resources that fit the needs of their patients. There were some concerns that they are not required to provide a survivorship plan anymore and all survivors may not need resources. Moreover, there were concerns about whether the tool would serve patients seen at other satellite clinics and concerns that working with COMPASS-CP was motivated because it is already being used by another part of the organization.

##### Implementation Climate: Compatibility

Health care professionals were asked how COMPASS-CP CFC fits with their values and norms, and the values and norms within the organization. Most participants said the tool fits in with their values to provide holistic wellness, best practices, and quality patient care. One participant commented as follows:

I definitely think it aligns with our organization’s mission and personal mission. I think it’s just to be able to fully address their holistic needs, to care for them beyond their immediate treatment, and to continue to take care of them related to side effects that they may have from long or late-term toxicities, it fits within the promise that we have to care for our patients.

Participant ID 004

##### Implementation Climate: Learning

Most health care professionals said the climate is conducive to trying new things to improve work processes, yet they also do not always have the time and energy to think about ways to improve. Considerations for implementation of COMPASS-CP CFC included the benefit to the patient, need for physician buy-in, and context (eg, if a lot of change has happened recently).

##### Readiness for Implementation: Leadership Engagement

Health care professionals suggested the best roles to champion the tool would be nurse navigators, survivorship clinic providers, and the survivorship clinic director. It would also be important to have buy-in from other hematology/oncology providers, front desk schedulers, and administrators. They thought it would help with implementation to present the tool to the team only when it is finalized and to follow-up with patients about usefulness or challenges with community resources.

### Phase 3: Assessing the Usability of COMPASS-CP CFC

The demographic characteristics of health care professionals (informatics professionals and health care providers; n=5) and patients (n=5) who engaged in usability testing for COMPASS-CP CFC are described in Tables 1 and 2. The average System Usability Scale score was above our target score of 70 for both health care professionals and patients (Table 3), indicating an overall high ease of use. Yet, the range of scores reported by health care professionals included values lower than the target score, indicating the additional need for improved usability. Participants also quantitatively reported high levels of acceptability, appropriateness, and feasibility for COMPASS-CP CFC (Table 3). The following qualitative feedback guided by the CFIR elaborates on intervention and individual characteristics to consider for improving usability and for other aspects of future implementation planning.

**Table 3:** Summary of quantitative usability metrics.

Scale and participant group		Score			
		Mean (SD)	Median	Minimum	Maximum
**System Usability Scale (range 0-100)^a^**					
	Health care professionals	71.0 (15.2)	70.0	50.0	90.0
	Patients	95.5 (2.1)	95.0	92.5	97.5
	Overall	83.3 (16.5)	91.3	50.0	97.5
**Acceptability of Intervention Measure (range 1-5)^a^**					
	Health care professionals	3.9 (0.3)	4.0	3.5	4.3
	Patients	4.6 (0.6)	5.0	3.8	5.0
	Overall	4.2 (0.6)	4.0	3.5	5.0
**Intervention Appropriateness Measure (range 1-5)^a^**					
	Health care professionals	4.1 (0.1)	4.0	4.0	4.3
	Patients	4.7 (0.4)	5.0	4.0	5.0
	Overall	4.4 (0.5)	4.1	4.0	5.0
**Feasibility of Intervention Measure (range 1-5)^a^**					
	Health care professionals	4.1 (0.3)	4.0	4.3	5.0
	Patients	4.9 (0.3)	5.0	3.8	4.5
	Overall	4.5 (0.5)	4.4	3.8	5.0

^a^Higher scores indicate more agreement with the measured construct.

#### Intervention Design Quality and Packaging

Health care professionals offered suggestions regarding training and support that would be needed to deliver COMPASS-CP. Some explicitly requested a video recording or a videoconference, whereas others preferred written materials to describe workflow instructions (1 person asked for both). They also suggested giving context for why the CP is important, including if it contributes to patient satisfaction.

Training and support suggestions for future implementation from patients were also provided. It was mentioned that COMPASS-CP was self-explanatory and would not require training. Additionally, providing a support phone number to talk with someone if needing help would be useful, and providing instructions from the onset to explain how the patient-completed measures will be used (to generate a CP) would be supportive. They also noted that older patients may need more support.

#### Individual Knowledge and Beliefs About the Intervention

Health care professionals indicated preferences to facilitate ease of use of COMPASS-CP in the EHR. For example, participants suggested saving a summary of the CP in a clinic note with a link for the full plan as part of a clinical encounter. They would like to be able to edit it, and therefore, a PDF file would not work well. Moreover, providers said they would not open a saved PDF file. They emphasized the importance of embedding the tool within the provider workflow and emphasized that a patient’s primary care provider would need to be copied on a CP. Another health care professional commented that COMPASS-CP could be used to prompt research in addition to the CP and clinical action.

Patients said it would be helpful to refer to the plan regularly (eg, monthly) to track their goals and progress. Putting this plan in writing would serve as a helpful reminder of their goals. In addition, having the plan in the medical chart would provide motivation not to ignore it.

#### Additional Usability Feedback

Both health care professionals and patients indicated that it would be important to limit the number of resources provided, so recipients would not be overwhelmed. To do this, health care professionals said there would need to be enough information included in the resource list for a provider to make recommendations. Patients would also like to have providers review the CP with them.

## Discussion

### Principal Findings

The adaptation of COMPASS-CP CFC was rated as usable by health care professionals and patients who were colorectal cancer survivors. COMPASS-CP provides flexibility to compile assessments from different locations (eg, PROs and EHR data) and provides tools to help with interpretation of the data collected. COMPASS-CP also links identified concerns with personalized community resources. Incorporating validated PROMIS measures is an important strength of COMPASS-CP CFC since this measurement system has many advantages, including its ability to function as both a screening tool and a tool for assessing clinical outcomes [51-53]. We also enhanced the original COMPASS-CP tool by successfully integrating REDCap as the patient-facing interface, and this is particularly important because REDCap streamlines our ability to send surveys to patients, which they can complete independently (as opposed to a facilitated telephone interview), and allows investigators to easily make modifications. It is also possible for patients to complete the assessments on paper and for responses to be entered by clinical staff.

The COMPASS-CP CFC tool facilitates integrating patient-completed assessments in clinical care, which is relevant to improving patient engagement and patient-provider communication [54,55]. Patient feedback in this study and other studies indicated that providers are expected to discuss the concerns raised in measures completed by patients and that this would influence the selection of the personalized recommended actions in the CP. COMPASS-CP CFC has the potential to reduce the time it takes providers to comprehensively assess and prioritize patient concerns during a clinic visit, thus facilitating more time for coaching to empower patients’ self-management. Supporting patients to increasingly engage in self-management and connecting them with relevant resources are important components of proposed systematic changes that involve risk stratifying the delivery of follow-up cancer care [56,57]. COMPASS-CP CFC could facilitate access and referral to recommended supportive interventions that are often not integrated into standard care.

Health care professionals indicated that the ability of COMPASS-CP CFC to link patients’ concerns with resources near their place of residence was particularly valuable since it was an unmet need that could potentially save them time. Yet, health care professionals were also concerned about fulfilling the responsibility to provide adequate and timely resources for the concerns identified (eg, a patient who scores high on concerns such as depression). Thus, we considered that it was particularly important for each patient-completed measure included in COMPASS-CP CFC to be linked to an actionable resource. The next steps may include integrating COMPASS-CP CFC with an existing national database of community resources that is increasingly used for addressing social determinants of health to support the feasibility of maintaining current and up-to-date resources [58]. This integration would further enhance scalability.

Incorporating stakeholder feedback in this adaptation process is a critical step to ensure that COMPASS-CP CFC will be successfully implemented in future work [59]. Data collected using the CFIR will particularly be applied to inform the integration of COMPASS-CP CFC into clinical care. As currently designed, for the use of COMPASS-CP CFC, it will be important to include a shared decision-making discussion with a health care professional when selecting and providing resources to ensure that patients’ concerns are normalized and appropriately prioritized [60]. Future research will explore the appropriate professional for this role. As suggested by a stakeholder, this professional may be a health coach or social worker who is integrated with clinical care. The appropriate professional may also vary based on the local clinical context. The next steps for this line of work include pilot testing COMPASS-CP CFC when integrated within clinical care to assess further usability and effectiveness. It will be important to assess the appropriate timing to implement COMPASS-CP CFC in the follow-up care process. Moreover, it will be important to consider how to follow-up with patients and encourage ongoing adherence to CPs.

### Limitations

A limitation of this study includes the range of usability scores reported by health care professionals, with some scores being below the target score. This indicates that further work is needed to improve usability. Future studies may incorporate additional usability feedback from health care professionals to further optimize COMPASS-CP CFC. In addition, COMPASS-CP CFC has not yet been tested in the flow of clinical care, which will be an imperative next step for tool refinement. Prior research on electronic care planning support interventions suggests that adopting COMPASS-CP CFC at multiple time points as part of a dynamic care planning process will result in the largest impact [15]. Furthermore, although this study was focused on follow-up care specific to colorectal cancer, we designed COMPASS-CP CFC to be easily adapted to other cancer types (by modifying the colorectal cancer–specific items). We chose to begin with colorectal cancer because these patients commonly have a good prognosis with a high need for follow-up care and this cancer type is prevalent in both men and women. Therefore, the next steps include broadening stakeholder involvement and considering patients diagnosed with other types of cancers.

### Conclusion

We gathered stakeholder feedback to inform our iterative adaptation of COMPASS-CP CFC to facilitate patient engagement in their follow-up cancer care. This electronic tool is integrated with the EHR and provides flexibility to comprehensively compile assessments from different locations (including PROs), add assistance with interpretation of the data collected, and link concerns identified with personalized resources. This study supports the initial usability of COMPASS-CP CFC and will inform the next steps for implementation in clinical care. COMPASS-CP has the potential to be a widely applicable and scalable tool for personalizing and streamlining comprehensive follow-up cancer care. The implementation of this tool in coordination with clinical care may ultimately facilitate sustainable improvements in patient health outcomes.

## Abbreviations

API: application programming interface
CFC: cancer follow-up care
CFIR: Consolidated Framework for Implementation Research
CP: care plan
EHR: electronic health record
FHIR: Fast Health Interoperability Resources
IDEAS: Integrate, Design, Assess, and Share
PRO: patient-reported outcome
PROMIS: Patient-Reported Outcomes Measurement Information System
SMART: Substitutable Medical Applications and Reusable Technologies

## Data Availability

The data sets generated during or analyzed during this study are available from the corresponding author upon reasonable request.
